# Aging-related aneuploidy is associated with mitochondrial imbalance and failure of spindle assembly

**DOI:** 10.1038/s41420-023-01539-2

**Published:** 2023-07-08

**Authors:** Fa-Li Zhang, Wei-Dong Li, Ke-Xin Zhu, Xu Zhou, Lan Li, Tin-Lap Lee, Wei Shen

**Affiliations:** 1 College of Life Sciences, Key Laboratory of Animal Reproduction and Biotechnology in Universities of Shandong, Qingdao Agricultural University, 266109 Qingdao, China; 2 College of Animal Science and Veterinary Medicine, Shandong Agricultural University, 271018 Tai’an, China; 3 Advanced Medical Research Institute, Shandong University, Jinan, China; 4 Center for Reproductive Medicine, Cheeloo College of Medicine, Shandong University, Jinan, China; 5 Developmental and Regenerative Biology Program, School of Biomedical Sciences, Faculty of Medicine, The Chinese University of Hong Kong, Shatin, Hong Kong China; 6 EggLoigcs Limited. Hong Kong Science and Technology Park, Shatin, Hong Kong China

**Keywords:** Ageing, Oogenesis

## Abstract

Despite aging is closely linked to increased aneuploidy in the oocytes, the mechanism of how aging affects aneuploidy remains largely elusive. Here, we applied single-cell parallel methylation and transcriptome sequencing (scM&T-seq) data from the aging mouse oocyte model to decode the genomic landscape of oocyte aging. We found a decline in oocyte quality in aging mice, as manifested by a significantly lower rate of first polar body exclusion (*P* < 0.05), and dramatically increasing aneuploidy rate (*P* < 0.01). Simultaneously, scM&T data suggested that a large number of differential expression genes (DEGs) and differential methylation regions (DMRs) were obtained. Next, we identified strong association of spindle assembly and mitochondrial transmembrane transport during oocyte aging. Moreover, we verified the DEGs related to spindle assembly (such as *Naip1, Aspm, Racgap1, Zfp207*) by real-time quantitative PCR (RT-qPCR) and checked the mitochondrial dysfunction by JC-1 staining. Pearson correlation analysis found that receptors for mitochondrial function were strongly positively correlated with abnormal spindle assembly (*P* < 0.05). In conclusion, these results suggested that the mitochondrial dysfunction and abnormal spindle assembly of aging oocytes ultimately may lead to increased oocyte aneuploidy.

## Introduction

During the past few decades, taking comprehensive factors such as education, society, and economy into consideration, the average age of newborn mothers worldwide has increased significantly [1]. In 1973, only 2% of women for the first time chose to give birth ranging the age from 35–44. However, in 2012 this number surprisingly reached 13%. Currently, the ratio continues to rise [2, 3]. However, it is not clear how age increases and decreased ovarian reserve affect the quality of oocytes.

Aneuploidy usually causes embryonic developmental arrest and implantation failure, resulting in spontaneous abortion of the fetus or birth of chromosomally unbalanced offspring [4], which is a fatal injury to the health of most animals, especially for older women. Aneuploidy is rare in males, about 2%, while numerical chromosomal aberrations in females are common, and about 20% of oocytes are abnormal, indicating the females are more impressionable to chromosomal errors [5]. Studies have shown that aging is one of the significant causes of oocyte aneuploidy, which can lead to female infertility [6]. A retrospective analysis of 15,000 embryo biopsies illuminated that among women older than 42 years old, 75%–100% of embryos are aneuploid; while at the age ranging from 26 to 30, the rate of aneuploidy was only 20–27% [7]. The chromosome segregation error of oocytes increases sharply with age, from less than 5% at the age of 3 months to as high as 30%–50% at the age of 12 months in mouse [4]. Thus, it can be seen that mice remain the best available and ideal model to explore the rationale of chromosome segregation in aging oocytes.

It is worth mentioning that the most abundant organelles in oocyte plasma are the mitochondria. From fertilization to blastocyst implantation, mitochondria no longer replicate in mature oocytes, indicating that mitochondria in the cytoplasm have a pivotal role in oocyte maturation, fertilization, and later embryo development [8, 9]. During the development and maturation of oocytes, mitochondria dynamically distribute around the spindle MTs to provide ATP [10–12] and calcium ions to promote the recruitment and assembly of intracellular organelles and cytoskeleton. Studies have pointed out that ovarian senescence is linked to abnormal mitochondrial function and structure [13].

The single-cell parallel methylation and transcriptome sequencing (scM&T-seq) simultaneously detects transcriptome and DNA methylation state in a single-cell resolution, importantly, it provides a better understanding of the state of the cells [14, 15]. In this study, we reinvestigated the characteristics of the transcriptome and DNA methylation from scM&T-seq data in naturally aging mouse oocytes as well as explained the mechanism of age-dependent aneuploidy at the genetic level. Moreover, we put forward that the abnormal mitochondrial function and metabolism of aging oocytes caused insufficient energy supply during the process of spindle recruitment and assembly, resulting in the spindle to collapse, and ultimately leading to the appearance of aneuploidy.

## Results

### Oocyte quality declines in aging mice

**Fig. 1:**
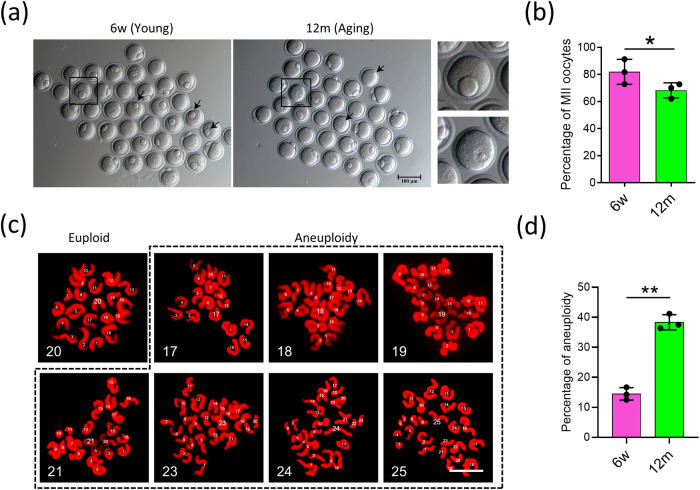
Quantifying oocyte quality in young and aging mice. **a** Representative images of young oocytes at 6 weeks (6w) and aged oocytes at 12 months (aging) after culturing for 12 h. **b** The rate of the 1st polar body extrusion in the two groups of oocytes. *n* = 3; Data were presented as mean ± SD; Compared to 6w group, * means *P* < 0.05. *T*-test was used to identify the difference. **c** Representative images of euploidy (20 univalents) and aneuploidy (less or more than 20 univalents) in MII oocytes. bar = 10 μm. **d** The rates of aneuploid oocytes in the young mice (6w) and aging mice (12 m); *n* = 3; Data were presented as mean ± SD; Compared to 6w group, ** means *P* < 0.01. *T*-test was used to identify the difference.

A hallmark of low-quality oocyte during aging is manifested by the low extrusion rate of the 1st polar body. To establish a reliable animal model to reflect oocyte aging, we first compared mouse MII oocytes from 6-week (6w) and 12-month (12 m) and examined the 1st polar body extrusion rate (Fig. 1a). The rate of first polar body exclusion in aging eggs decreased by about 15% (Fig. 1b), suggesting the aging has negative impact on oocyte quality. Figure 1c shows representative images of chromosome spread in euploid (20 univalents) and aneuploid (less or more than 20 univalents) oocytes from young and aged mice. MII oocytes from aged mice showed a significantly higher incidence of aneuploidy (38.3 ± 2.5%) than oocytes from young mice (14.5 ± 2.1%) (Fig. 1d; *P* < 0.01).

### The transcriptome and methylation data landscape of mouse oocytes

**Fig. 2:**
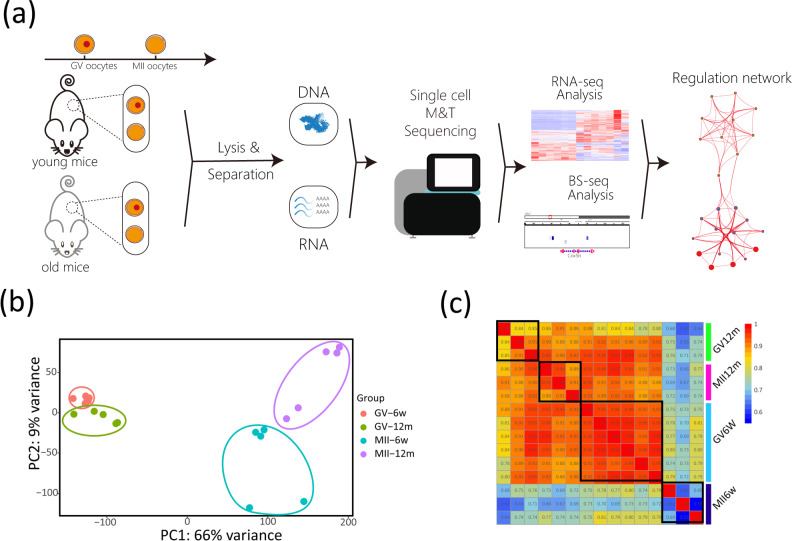
The transcriptome and methylation data landscape of mouse oocytes. **a** The brief workflow of bioinformatic analysis. **b** The PCA analysis of single-cell RNA-seq data. **c** The heatmap showed the Pearson correlation coefficient of single-cell Bisulfite-seq data.

To proceed to genomic level analysis, scM&T-seq was performed to obtain transcriptome and DNA methylation data for both immature (GV) and mature (MII) stages from young (6w) and aging (12 m) mice. Here, a brief analysis and experiment workflow was presented (Fig. 2a). The principal components analysis of samples (PCA) and the euclidean distances analysis of samples in single-cell RNA-seq showed that the transcriptome we obtained has good repeatability within the group, and the transcripts of GV phase and MII phase were quite different (PC1: 66% variance) (Fig. 2b and S1A). Simultaneously, the Pearson correlation coefficient analysis of single-cell Bisulfite-seq showed that four distinct modules were obtained, confirming the repeatability within the sample group (Fig. 2c).

### Identification of differences between the young and aging groups

**Fig. 3:**
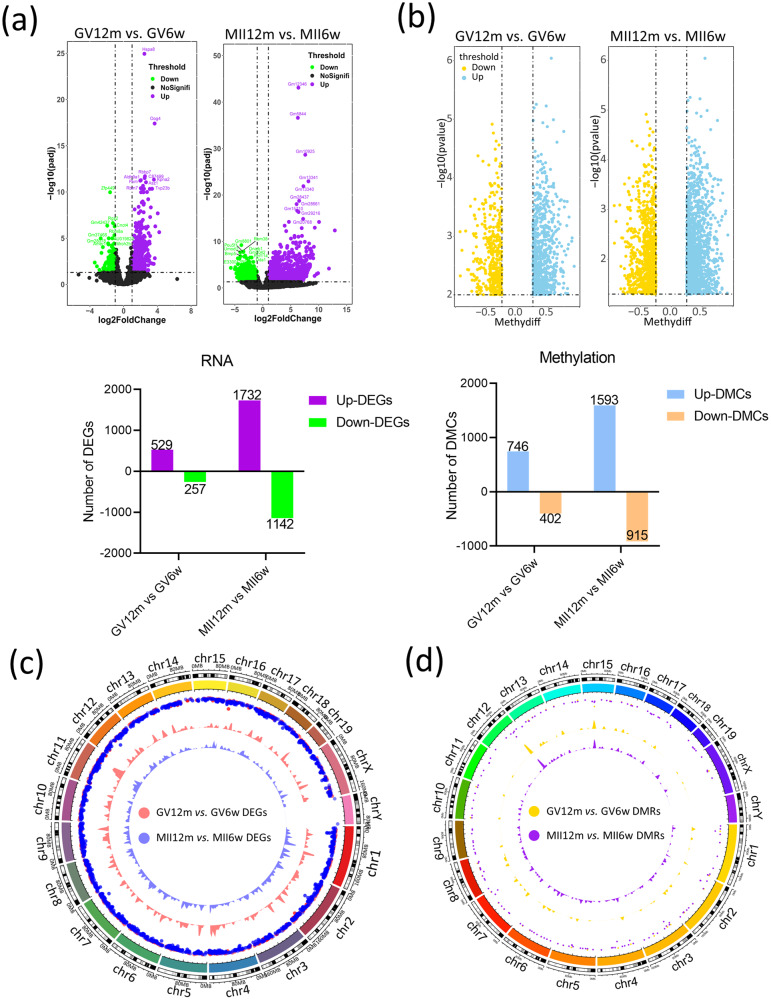
Identification of differences between the young group and the aging group. **a** The Volcano Plot showed DEGs in GV12m vs. GV6w group (up left) and MII12m vs. MII6w group (up right), and the Bar Chart (bottom) showed the statistics results. **b** The Volcano Plot showed DMCs in GV12m vs. GV6w group (up left) and MII12m vs. MII6w group (up right), and the Bar Chart (bottom) showed the statistics results. **c** The Chord Diagram was adopted to visualize the distribution and expression of each DEGs in the chromosome. **d** The Chord Diagram was adopted to visualize the distribution and expression of each DMRs in the chromosome. DEGs differential expression genes. DMCs differentially methylated cytosines. DMRs differentially methylated regions.

Next, we pairwise compared each set of samples, including GV oocytes produced from 12-month-old mouse (GV12m) vs. GV oocytes produced from 6-week-old (GV6w), MII oocytes produced from 12-month-old mouse (MII12m) vs. MII oocytes produced from 6-week-old (MII6w). For the above four comparisons, we obtained 786 (up left) and 2873 (up right) DEGs in turn (Fig. 3a). For DNA methylation data, firstly we identified differential methylation cytosines (DMCs) and differential methylation regions (DMRs). The profiles of DMCs in the different group showed in Fig. 3b. The DMCs in GV stage was 1148 (up left), however, in MII stage was reached to 2508 (up right) (Fig. 3b). Moreover, the DMRs in different group was visualized via chord diagram (Fig. 3d). In chromosome 12 and chromosome 15 a peak occurred (Fig. 3d), meanwhile the low distribution and expression in transcriptome data was observed (Fig. 3c).

**Fig. 4:**
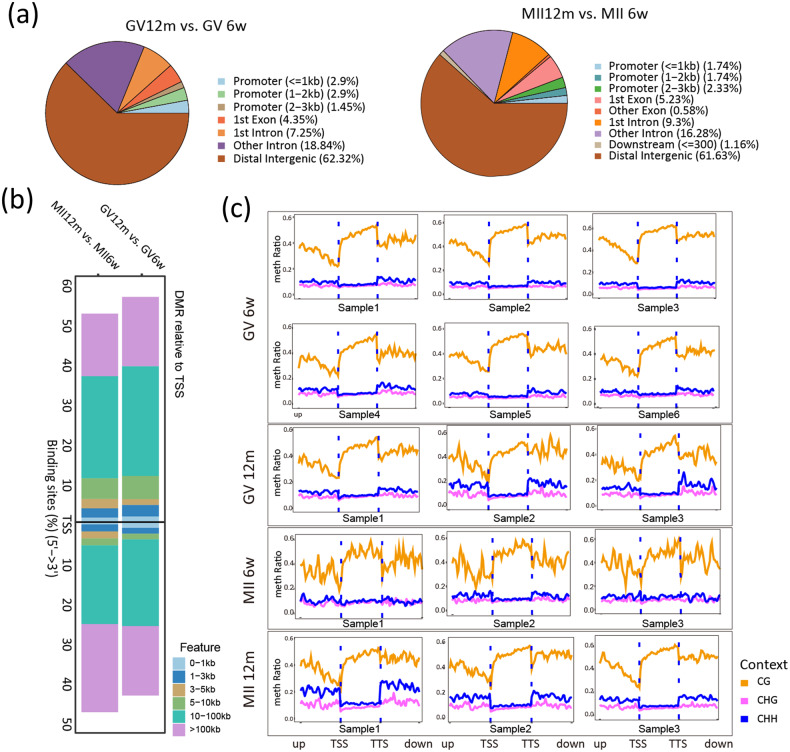
Methylation data visualization. **a** The location of DMRs in the chromosome. **b** The location of DMRs relative to TSS. **c** The methylation levels of each sample, including three cytosine context, CG, CHG and CHH. TSS transcription start site. TTS transcription terminal site. The outer boxes represent different groups, and each small panel inside represents each sample.

To analyze the change of epigenetic landscapes, we annotated the DMRs in detail, including the location of DMRs in chromosome and the location of DMRs relative to transcription start site (TSS). Both in GV and MII stage, vast majority of DMRs located on distal intergenic, in GV stage was 62.23% and in MII stage was 61.63% (Fig. 4a). Specifically, the DMRs located in <3 kb promoter region only less than 10%, indicating that except for DNA methylation, other regulatory way plays a crucial role in aging progress. Sure enough, the location of DMRs relative to TSS in >10 kb region was vast majority (Fig. 4b). In addition to the above analysis, the methylation level of each sample also visualized by Fig. 3c, including three cytosine content, CG, CHG and CHH (where H stands for A or T or C). We observed that the methylation ratio of CHG or CHH increased in the aging group, especially in MII stage (Fig. 4c).

### Functional annotation of DEGs and genes related to DMRs

**Fig. 5:**
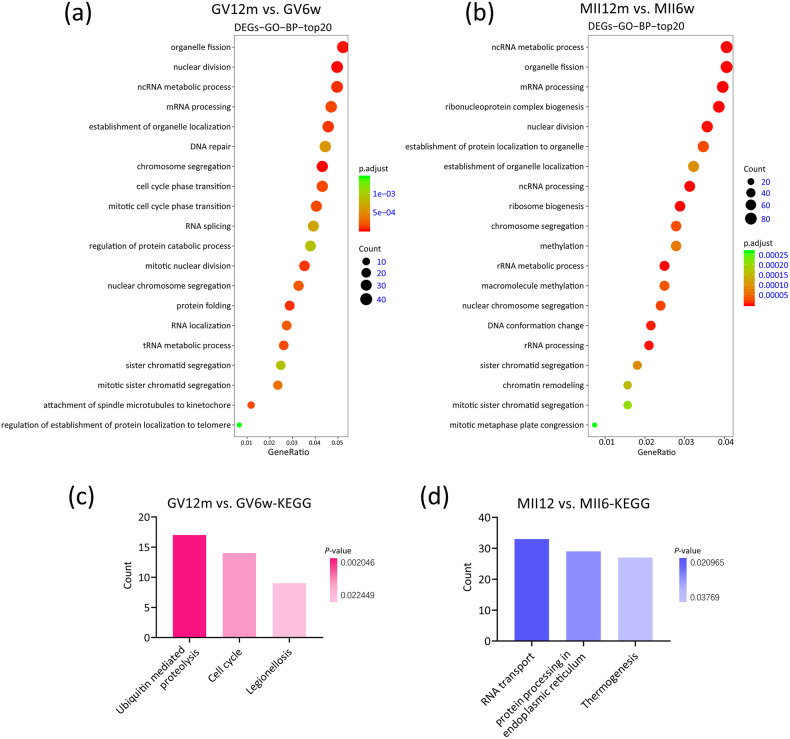
The function annotation of DEGs. **a** The top 20 GO-BP terms of DEGs in the GV12m vs. GV6w group. **b** The top 20 GO-BP terms of DEGs in the MII12m vs. MII6w group. **c** The KEGG terms of DEGs in the GV12m vs. GV6w group. **d** The KEGG terms of DEGs in the MII12m vs. MII6w group.

To identify the functional impact of DEGs, we applied gene ontology approach to reveal the functional association. In GV stage, aging oocytes are associated with changes in metabolic pathways, such as ncRNA metabolic process (GO:0034660), and cell cycle-related pathway, such as DNA repair (GO:0006281) (Fig. 5a). As expected, we also found spindle assembly (GO:0051225) on the list (Table S1). During the mature stage (MII), ncRNA metabolic process was highlighted (Fig. 5b), which is followed by mitochondrial transmembrane transport (GO:1990542) and intrinsic apoptotic signaling pathway in response to oxidative stress (GO:0008631) (Table S2).

Moreover, we observed changes of KEGG signaling pathways. In GV stage, the KEGG signaling pathway showed the enrichment of cell cycle (mmu04110) (Fig. 5c). In MII stage, another significant pathway, thermogenesis (mmu04714) was stand out (Fig. 5d).

### Collapse of spindle assembly

**Fig. 6:**
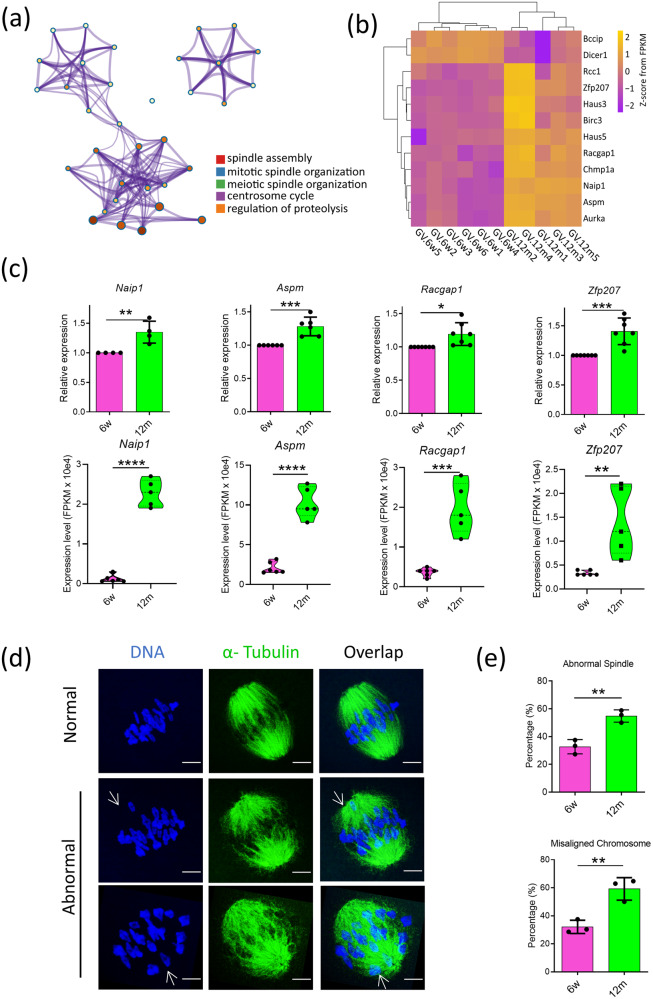
The aberrant spindle assembly. **a** The GO-BP annotation of the DEGs in spindle assembly terms. **b** The heatmap showed the trends of the DEGs in spindle assembly terms. **c** The relative expression of spindle assembly related genes, including *Naip1*, *Aspm*, *Racgap1* and *Zfp207* by RT-qPCR (upper panel; *n* > 3; Data were presented as mean ± SD; Compared to 6w group, * means *P* < 0.05, ** means *P* < 0.01, *** means *P* < 0.001. *T*-test was used to identify the difference) and RNA-seq (below panel; The FPKM values was used to showing expression level; *n* > 3; Compared to 6w group, ** means *P* < 0.01, *** means *P* < 0.001, **** means *P* < 0.0001. *T*-test was used to identify the difference). **d** Representative pictures of spindle morphologies and chromosome alignment in the young and old groups. Spindle (green) and chromosome (magenta). Scale bar = 10 μm. **e** The rates of aberrant spindles (up) and misaligned chromosomes (bottom) in young and old groups; *n* = 3; Data were presented as mean ± SD; Compared to 6w group, ** means *P* < 0.01. *T*-test was used to identify the difference.

Spindle assembly was annotated in GV stage of aging oocytes. For exploring the details in spindle assembly, firstly we took a reanalysis of GO annotation of spindle assembly (Fig. 6a) and observed the expression trends of genes (Fig. 6b). The results indicated that in GV stage of aging oocytes, spindle assembly was disordered. To verify our bioinformatics analysis, we adopted real-time quantitative PCR (RT-qPCR) to observe the transcriptome, including *Naip1*, *Aspm*, *Racgap1*, *Zfp207*. The results showed that in GV stage of aging oocytes this transcriptome was all over-expressed and it is consistent with the sequencing results, which indicated that the sequencing results were trustworthy (Fig. 6c). Moreover, we also observed the spindle morphology and chromosome arrangement through immunofluorescence microscopy (Fig. 6d). As expected, we found that the ratio of the aberrant spindle to the misaligned chromosome increased significantly in aging oocytes (Fig. 6e). Besides, the methylation signal of above DEGs, including *Naip1*, *Aspm*, *Racgap1*, *Zfp207*, was increased in the young group (Fig. S1) indicating that aging caused spindle regulation disorders and abnormal assembly.

**Fig. 7:**
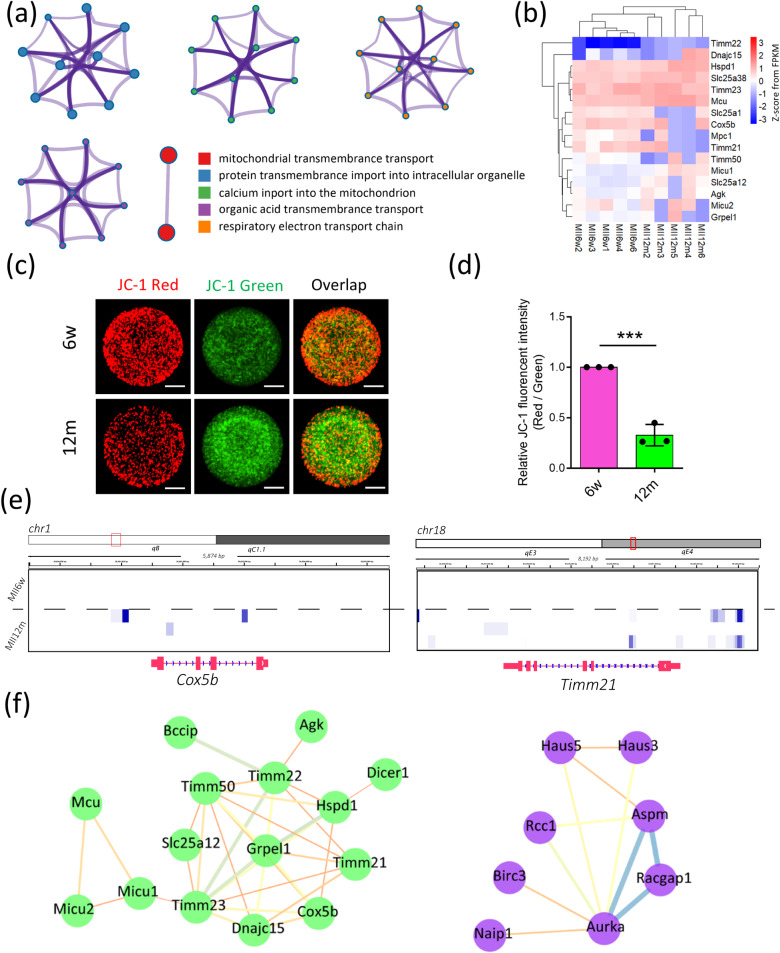
Briefly mitochondrial functional verification. **a** The GO-BP annotation of the DEGs in mitochondrial transmembrane transport terms. **b** The heatmap showed the trends of the DEGs in mitochondrial transmembrane transport terms. **c** Mitochondrial membrane potential was calculated as the fluorescence ratio of red/green, where red fluorescence corresponded to activated mitochondria (J-aggregates), and green fluorescence corresponded to less-activated mitochondria (J-monomers). Scale bar = 20 μm. **d** The relative fluorescence intensity of the mitochondrial membrane potential in the young and old groups; *n* = 3; Data were presented as mean ± SD; Compared to 6w group, ** means *P* < 0.01. *T*-test was used to identify the difference. **e** The methylation signal related to DEGs of mitochondrial transmembrane transport in each sample. Dark blue represented for the aging group. **f** The protein–protein interaction (PPI) network of DEGs in mitochondrial transmembrane transport terms (left), right is spindle assembly.

Another significant change in aging oocytes was mitochondrial function dysfunction in MII stage of aging oocytes in annotation terms. Similarly, we adopt a reanalysis of GO annotation of spindle assembly (Fig. 7a) and observed the expression trends of genes (Fig. 7b). In order to further verify the decline of mitochondrial function in aging oocytes and to support the sequencing results, we detected the changes in mitochondrial membrane potential by staining JC-1 (Fig. 7c). The results indicated that the mitochondrial membrane potential was significantly lower than that of normal oocytes (Fig. 7d). Besides, the methylation signal of above DEGs related to mitochondrial function dysfunction was increased in the aging group (Fig. 7e). The STRING database was selected to explore the protein–protein interaction network, the results showed that the *Timm* family protein was a key protein in the mitochondrial function dysfunction, we believed that *Timm* family protein played a core role in regulating mitochondrial function (Fig. 7f). Another protein–protein interaction (PPI) network showed that *Aspm, Aurka, Racgap1* were key protein in regulating spindle assembly, we considered that the abnormal expression of the above transcriptome results in low quality of aging oocytes (Fig. 7f).

KEGG function are the most valid way to show the metabolic pathway, two important metabolic pathways are selected to explore in detail, one for cell cycle and one for thermogenesis. We attempted to explore the details of these two pathways, and therefore reanalyzed them using Metascape. In GV stage, the cell cycle signaling pathway changed, and results showed that the G2/M checkpoint was activated, which was related to abnormal spindle assembly (Fig. S2A). In MII stage, based on our results we believed that mitochondrial function was subject to damage. We can see that one of the most obvious signaling pathways is oxidative phosphorylation (KEGG: mmu00190), which was consistent with mitochondrial dysfunction (Fig. S2B), although this data is not convincing enough, it suggested the possibility of the collapse of spindle assembly.

## Discussion

Aneuploidy of germ cells tends to lead to pregnancy loss by miscarriage or stillbirth, causing infertility, and which became one of the scientific problems of wide concern. There are many reasons for aneuploid changes, one of the important factors is abnormal spindle assembly. Although two models, "cohesion-loss" and "spindle collapse", have been suggested to illustrate the reasons for the increase in age-related aneuploidy [16], the underlying molecular mechanisms are mysterious as yet. Studies have shown that mice are a suitable model for studying age-dependent aneuploidy effects [17] because they can overcome the defects of suboptimal human oocyte quality and a wide age range [18]. Here, with aging mouse model, we conducted a joint analysis of the results of scM&T-seq of mouse oocytes in the GV and MII stages from the 6-week young group and 12-month aging group to identify age-related methylation modifications and gene expression profiles aiming to better explain the mechanism of aneuploidy caused by aging at the molecular level. Bioinformatics data have found that mitochondrial function is seriously disturbed during aging, which is manifested in the increase of ROS level and the disordered expression of related genes (such as *Cox5b, Timm21*). Interestingly, the Pearson correlation analysis showed that above was closely related to abnormal spindle assembly (r > 0.85, *P* < 0.05). Hence, we daringly hypothesize that oocyte aneuploidy may occur in older mice due to mitochondrial dysfunction and abnormal spindle assembly.

In 2017, research by Nakagawa and FitzHarris showed that in relatively older oocytes, the spindles responsible for the arrangement and separation of chromosomes during cell division will be abnormal. MTs that change their normal trajectory can cause errors in chromosome segregation, leading to aneuploidy [16]. The researchers collected oocytes from young mice aged 6 to 12 weeks and old mice aged 60 weeks and performed a series of microscopic analyses. The results showed that about 50% of oocytes had spindle defects. The researchers swapped the nuclei of young and old oocytes, but found that the old oocytes with the young nuclei were still defective, which suggested that the mother’s age interfered with the MTs arrangement, regardless of the age of the nucleus of the oocyte [16]. Recent studies have been indicated that age-dependent PSSCs may be caused by the gradual loss of the cohesion complex that maintains the unification of sister chromosomes and kinetochores in mouse and human oocytes [19–22]. However, the "non-disjunction" cannot be explained well by the "cohesion-loss" model, because the misaligned attachment of MTs-kinetochores in meiosis I of adult mouse oocytes is very weakly correlated with loss of cohesion [23], and so are the lagging anaphase chromosomes [24]. Therefore, "cohesion-loss" may not be the only senescence-related cell change that causes the error of chromosome separation in oocytes. In paralleling with this view, classic studies on human oocytes have observed defects in the spindle morphology of oocytes in elderly women, suggesting that age may exert a negative effect on MTs [25, 26]. Unfortunately, so far, the related molecular mechanism and the cause of the spindle defect are still not clear.

In this study, our results showed that whether it was gene expression profile or methylation modification, greater differences were observed in the young group and the old group both in the GV stage and the MII stage. Based on this, we used GO and KEGG enrichment analysis to functionally annotate the above-mentioned DEGs (Fig. 5) and found that these DEGs are mainly associated with the assembly of spindle MTs and the metabolism of mitochondrial functions. Next, we used the heatmap to visualize some DEGs related to the spindle assembly. Further, four of the genes, including *Aspm*, *Racgap1*, *Naip1*, and *Zfp207*, were selected to experimentally verify by RT-qPCR, and as expected the results of RT-qPCR were highly consistent with the sequencing analysis, indicating the sequencing data was convincing. In addition, through immunofluorescence microscopy, we observed the spindle morphology and chromosome arrangement of oocytes of the two groups, and also calculated the proportion of abnormal spindle and misaligned chromosome. The results showed that the abnormal spindle and misaligned chromosome of oocytes in the old group were significantly higher than those in the young group. In order to explore why these genes are highly expressed in aging oocytes, we analyzed the related epigenetic modification status, and found that there are lower levels of methylation modification sites in the oocytes of the aging group. As we all know, DNA methylation, as a form of DNA chemical modification, usually inhibits gene transcription [27]. Therefore, the highly expressed genes in these aged oocytes may be caused by fewer methylation modifications. Next, we are very interested in what causes the defect of the spindle, and we have further explored.

Mitochondria are important organelles in eukaryotic cells and are the main participants in energy metabolism and ATP production [28]. Recent studies have shown that the normal function of mitochondria is the guarantee of the quality of oocytes and the potential of embryonic development, while the senescence of oocytes is linked to mitochondrial dysfunction and energy metabolism disorders [29–31]. Mitochondrial DNA (mtDNA) damage, dysregulation of mitochondrial gene expression and decrease of mitochondrial membrane potential (MMP) may all be the causes of mitochondrial dysfunction in oocytes of elderly women [32]. As shown in Fig. 7c, through JC-1 staining, we first detected the changes in MMP. As expected, the MMP of oocytes in the old group significantly decreased. The normal MMP is the prerequisite for maintaining mitochondrial oxidative phosphorylation to produce ATP. Therefore, the decreased mitochondrial membrane potential may cause a decrease in ATP production, resulting in insufficient energy supply in the progress of spindle recruitment and assembly, and ultimately leading to the appearance of aneuploidy. Therefore, we suggested that the defective spindle that causes aneuploidy in aging oocytes may be due to the decreased ATP production caused by mitochondrial dysfunction. Besides, in order to understand the reasons for the decline in mitochondrial function, we then analyzed the gene expression and epigenetic modification in the two groups of cells. The results revealed that the expression of some genes in the old group, such as *Cox5b*, *Hspd1*, *Slc25a38*, *Mcu* and *Timm21*, was significantly lower than that in the young group (Fig. 7b). Expectantly, the methylation sites of down-regulation DEGs were higher in aging oocytes, more interestingly no methylation site signals were found for these DEGs (Fig. 7e). Based on the above, we speculate that the high abundance of methylation has caused the expression of mitochondrial-related genes in aging oocytes to decrease, which causes the disorder of mitochondrial function.

**Fig. 8:**
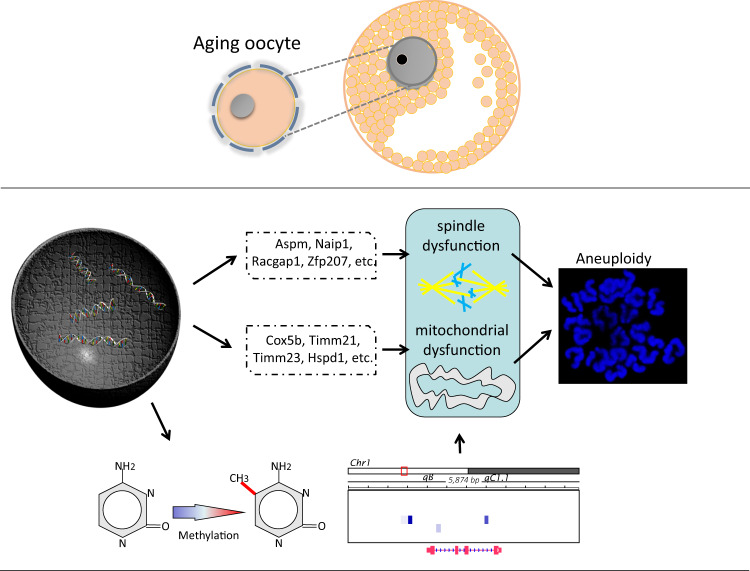
The summarization of an explanation of aneuploidy in the genetic level. Upper represents an aging oocyte and below are the differences in the genetic level, including transcriptome and DNA methylation.

In conclusion, our study provided new evidence that supports the link between age and aneuploidy. We have identified defective spindles as the underlying mechanism that leads to aneuploidy at a molecular level (Fig. 8). Our findings suggest that as oocytes age, the mitochondrial function and metabolism become abnormal, resulting in inadequate energy supply during spindle recruitment and assembly. This insufficient energy supply causes spindle collapse and ultimately leads to aneuploidy. Our conclusion adds to the molecular understanding of why there is an increase in age-related aneuploidy among women.

## Experimental procedures

### Oocyte collection and culture

Female mice of CD1 background were obtained from Vital River Laboratory Animal Technology Co. LTD (Beijing, China), and eat and drink freely in a 23 °C temperature-controlled room with a 12 h light/dark cycle. All experimental protocols were approved by the Experimental Animal Ethics Committee of Qingdao Agricultural University. Pregnant mare serum gonadotropin (PMSG) was injected firstly, 6 weeks, and 12 months old CD1 female mice were euthanized after 48 h and subsequently ovaries were collected in M2 medium supplemented with 2.5 μM milrinone. And then oocytes were picked up and about 30 oocytes in GV stage of each group were put into 30 μL of M16 medium droplets covered with mineral oil and in vitro matured at 37 °C in a 5% humid CO_2_ atmosphere. After 12 h of culture, we observed and counted the number of oocytes extruded the 1st polar body under a stereo microscope, and calculated the maturity rate.

### Chromosome spread staining

Chromosome spread staining was performed as previously described [33]. In a short, oocytes were removed from the zona pellucida by exposure to acidic Tyrode’s solution (pH = 2.5). Then, fix in 1% PFA in distilled water (pH = 9.2) containing 0.15% Triton X-100 and 3 mM dithiothreitol. After air drying, chromosomes were counterstained with propidium iodide (PI, E607306; Sangon Biotech) and visualized with an inverted fluorescence microscope (IX71, Olympus).

### Immunofluorescence and confocal microscopy

Immunofluorescence staining was performed as previously described [33–35]. Briefly, the oocytes were fixed in 4% paraformaldehyde at room temperature for 0.5 h. Next, the oocytes were permeabilized for about 20 minutes in D-PBS solution supplemented with 0.5% Triton X-100 and blocked for 1 h in D-PBS containing 1% bovine serum albumin (BSA). The oocytes were then incubated in the anti-α-tubulin antibody (1:500; SC-5286; Santa Cruz Biotech, TX, USA), overnight at 4 °C. Subsequently, the oocytes were incubated for 1 h at room temperature in the FITC conjugated IgG (1:1000; Sangon Biotech, Shanghai, China). The DNA was counterstained with DAPI (C1022; Beyotime, Shanghai, China) for about 10 min. Finally, use appropriate fluorescent anti-quenching agents to seal and the images were captured by a laser-scanning confocal microscope (Leica TCS SP5 II, Mannheim, Germany).

### RNA extraction and real-time quantitative PCR (RT-qPCR)

The SPARKeasy Tissue/Cell RNA Rapid Extraction Kit (Shandong Sparkjade Biotechnology Co., Ltd., AC0202, China) was selected to extract oocytes mRNA. Next, we used HiScript II Q RT SuperMix (Vazyme Biotech, Nanjing, China) to reverse-transcribed mRNA to cDNA. In the ABI 7500 Sequence Detection System, AceQ qPCR SYBR Green Master Mix (Vazyme) was used for RT-qPCR. Of note, amplification was performed in 20 μL reactions according to manufacturer’s instructions. The amplification conditions of PCR were as follows: the reaction was initialed at 95 °C for 5 min, followed by 40 cycles of denaturing at 95 °C for 10 s, annealing at 60 °C for 34 s and extension at 72 °C for 20 s. The ^2-△△Ct^ method was used to calculate the relative gene expression to *β-actin* [36, 37]. The primer sequence is detailed in Table S3.

### Mitochondrial membrane potential (Δψm) assay

The detection of Δψm was performed as previously described [38]. In short, the oocytes, after washing with PBS, incubated in a medium containing 0.5 μmol/L JC-1 (Invitrogen, Grand Island, NY, USA) at 37 °C in 5% CO_2_ for 0.5 h. The Δψm was showed with the fluorescence ratio of red/green. The red represented to activated mitochondria (J-aggregates), and the green represented to less-activated mitochondria (J-monomers).

### Single-cell M&T sequencing

A modified single-cell M&T-seq protocol for generating simultaneous transcriptome and methylome profiles from single oocytes [15]. Briefly, single oocytes are lysed using a soft lysis buffer [500 mM KCl (Sigma), 100 mM pH 8.0 Tris-HCl (Sigma), 1.35 mM MgCl2 (Sigma), 4.5 mM DTT (Thermo Fisher Scientific), 0.45% Nonidet P-40 (Sigma), 0.18 U SUPERase-In (Thermo Fisher) and 0.36 U recombination Naseinhibitor (Thermo Fisher)] and incubated at 4 °C for 30 min. The supernatant was used for SMART-seq2 library construction by using the TruePrep DNA Library Prep Kit V2 for Illumina (Vazyme) kit. The remaining DNA was bisulfite converted, amplified, and scBS-seq library construction using the Pico Methyl-Seq™ Library Prep Kit (Zymo Research). Qualified libraries were multiplexed and sequenced on Illumina HiSeq2500 platform for generating 150 bp paired-end reads.

### Single-cell RNA sequencing analysis

The single-cell RNA-seq data firstly had performed quantity controlling by FastQC (v0.11.8), then data of below the mark was filtered. The remaining part of single-cell RNA-seq data has performed another process that Fastp (v0.19.5) was adopted to scan the quantity of data [39]. In addition, we used BBmap to filter overrepre`sented sequence, and then clean data were obtained [40]. STAR (v2.7) (https://github.com/alexdobin/STAR) was selected to mapping to the genome (GRCm38.p5), except for “—outSAMtype BAM SortedByCoordinate”, the other parameter is the default.

### Different expression analysis

FeatureCounts (v1.6.4) was used to count reads of genes, further we used DESeq2 to determine the difference of mRNAs [41, 42]. Finally, the standard thresholds were *p*_adj_ < 0.05 and |log2FoldChange | > 1, when mRNAs met the above conditions, they were considered as differential expression mRNAs. We believed that differentially expressed mRNAs had an important function and they would perform follow-up analysis [34]. All expression of mRNAs in this paper adopts transcripts per kilobase million (TPM) method to normalize.

### Functional enrichment analysis

Gene ontology (GO) and kyoto encyclopedia of genes and genomes (KEGG) signaling pathway analysis were a powerful tool for exploring functional annotations of genes. Liking what we did before, the differential expression mRNAs in this study were performed above analysis. ClusterProfiler (v3.8.1) and Metascape (v11.0, http://metascape.org) were employed to perform GO and KEGG pathway analysis [43, 44]. Notably, the critical value of valid GO terms was *P* < 0.01, and KEGG signaling pathway was *P* < 0.05.

### Protein–protein interaction (PPI) analysis

STRING (v11.0, http://string-db.org/) was selected to perform PPI analysis, containing the interacted relationship, directly or physically related, between proteins and genes. Moreover, we used the tools of MCODE, Cytospace (v3.7.2) to show the relationship between differently expressed mRNAs and proteins [45].

### Single-cell bisulfite sequencing analysis

The single-cell Bisulfite-seq data firstly were performed quantity controlling by FastQC (v0.11.8), then data of below the mark was filtered [46]. BatMeth2, a powerful and excellent tool to deal with methylation data, is used to analyze the remaining part of single-cell Bisulfite-seq data [47]. Briefly, the genome index (GRCm38.p5) was built via “build_index” mode. Subsequently, the raw reads adopt “pipel” mode to produce methylation results, of note the “--fastp” parameter was specified. Then, we adopted “auto mode” to find DMCs and DMRs and the “-maxdis 10000” parameter was specified. Finally, we selected “methyPlot” mode to visualize methylation data. Other than the parameters indicated, the rest is the default. It should be noted that we used the CG values of “sample.methylevel.1.txt” produced from BatMeth2 in each sample to calculate the correlation.

### Statistical analysis

It is necessary to repeat the experiment at least three times and we used mean ± standard deviation (SD) to show the results, moreover Student’s *t*-test with the *P* < 0.05 via Graphpad Prism 8.0 software was adopted to identify differences among samples.

## Supplementary information

supplementary figure legends

FigS1

FigS2

Table S2

Table S3

Table S1

## Data Availability

All scM&T-seq data of mouse oocytes has been deposited in NCBI GEO database (GSE119906).
