# Supplementary material for: Aging-related aneuploidy is associated with mitochondrial imbalance and failure of spindle assembly
Source: Cell Death Discov. 2023 Jul 8;9:235. doi: 10.1038/s41420-023-01539-2 (PMC10329675; doi:10.1038/s41420-023-01539-2)
Supplement: Supplementary file 1 — supplementary figure legends [file 41420_2023_1539_MOESM1_ESM.docx]

**Figure S1.** Supplementary DNA methylation analysis for supporting Figure 5. The methylation signal related to the above DEGs in each sample. Pink for the young group, dark green for the aging group, orange represents the hypermethylated signal in the young group.

**Figure S2. Supplementary KEGG analysis for supporting Figure 5 and Figure 6**. A, The KEGG annotation of the DEGs in spindle assembly terms. B, The KEGG annotation of the DEGs in mitochondrial transmembrane transport terms.
