# Supplementary figures and images for: Aging-related aneuploidy is associated with mitochondrial imbalance and failure of spindle assembly
Source: Cell Death Discov. 2023 Jul 8;9:235. doi: 10.1038/s41420-023-01539-2 (PMC10329675; doi:10.1038/s41420-023-01539-2)

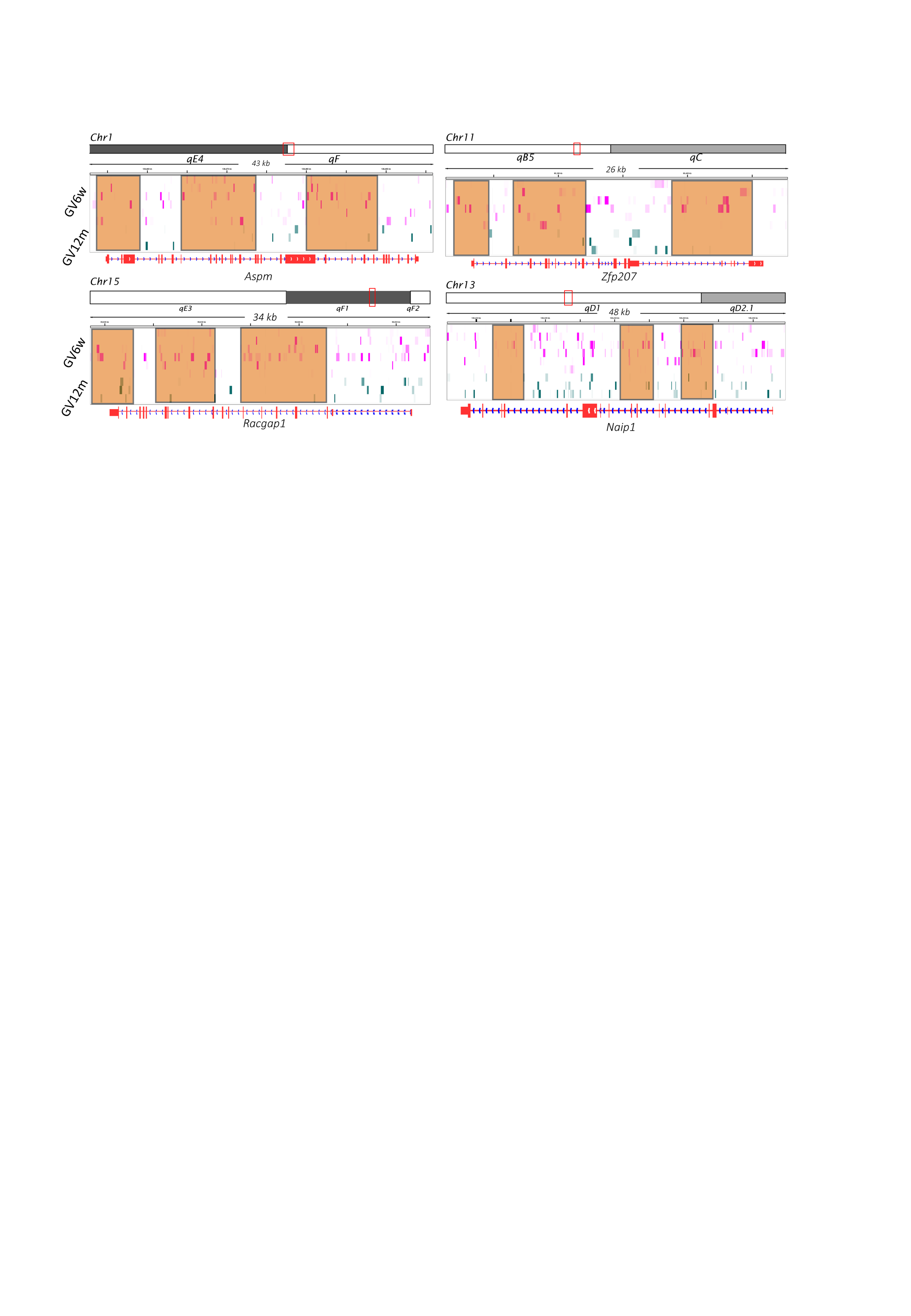

Supplement: Supplementary file 2 — FigS1 [file 41420_2023_1539_MOESM2_ESM.tif]

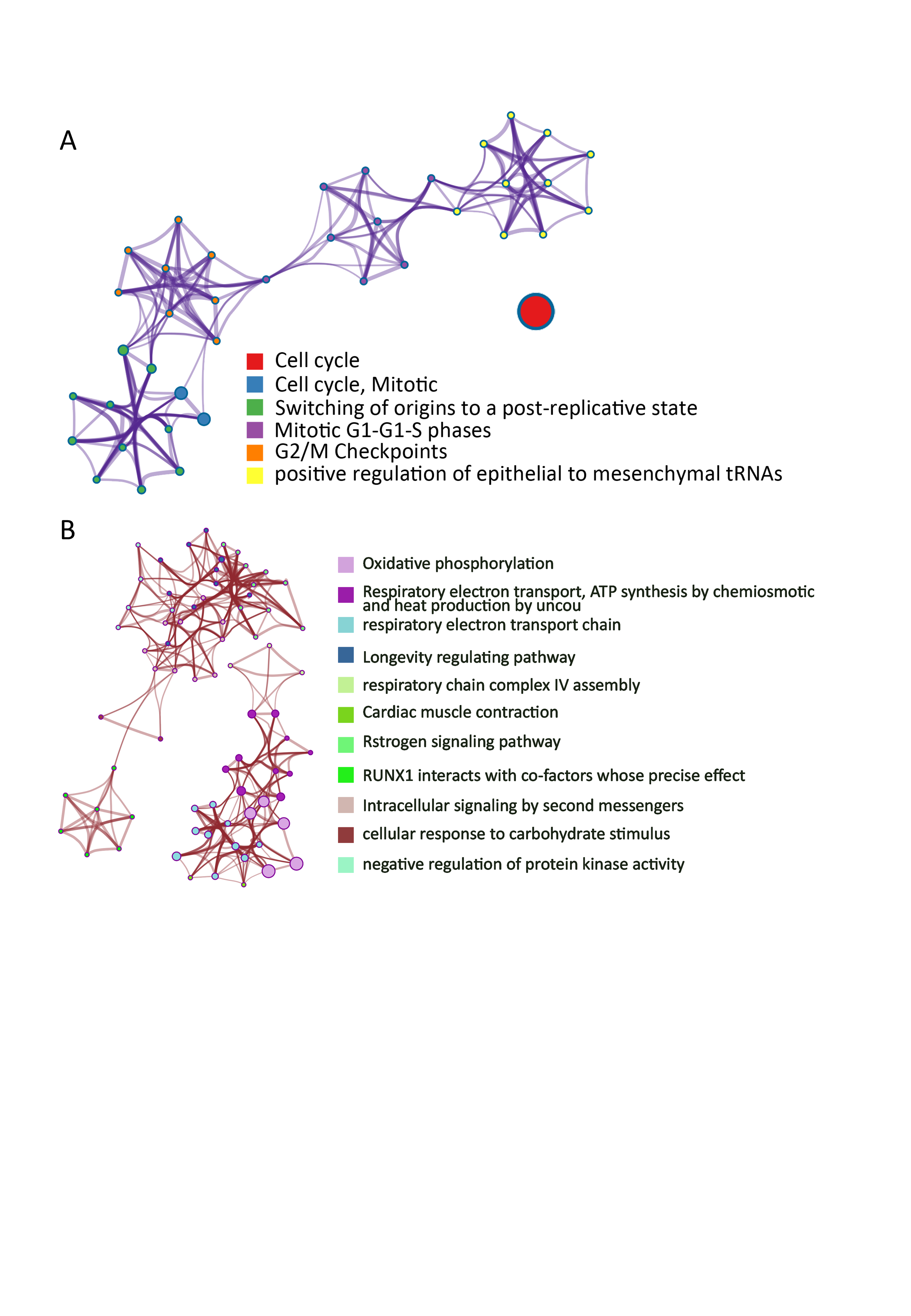

Supplement: Supplementary file 3 — FigS2 [file 41420_2023_1539_MOESM3_ESM.tif]
