# Supplementary material for: Aging-related aneuploidy is associated with mitochondrial imbalance and failure of spindle assembly
Source: Cell Death Discov. 2023 Jul 8;9:235. doi: 10.1038/s41420-023-01539-2 (PMC10329675; doi:10.1038/s41420-023-01539-2)
Supplement: Supplementary file 6 — Table S1 [file 41420_2023_1539_MOESM6_ESM.docx]

**Table S1. Primer sequences**

| Gene | Sequence |
| --- | --- |
| *Naip1-F* | TGCCCAGTATATCCAAGGCTAT |
| *Naip1-R* | AGACGCTGTCGTTGCAGTAAG |
| *Aspm-F* | TGGCTATGAGTGAATGCTCTTCC |
| *Aspm-R* | TCGCGTAAAAACAGTGGCAAG |
| *Racgap1-F* | CGCCGGATGGAGATTATCAATG |
| *Racgap1-R* | CCCCGTCTCTGCTTTCAACAA |
| *Zfp207-F* | AGCCTGCTACACTCACAACC |
| *Zfp207-R* | TGGCATTGGAGGTCGCATT |
